# Preventive Effect of Greater Occipital Nerve Block on Severity and Frequency of Migraine Headache

**DOI:** 10.5539/gjhs.v6n6p209

**Published:** 2014-07-29

**Authors:** Davood Kashipazha, Ali Nakhostin-Mortazavi, Seyyed Ehsan Mohammadianinejad, Mohammad Bahadoram, Sepideh Zandifar, Shahram Tarahomi

**Affiliations:** 1 Department of Neurology, Ahvaz Jundishapur University of Medical Sciences, Ahvaz, Iran; 2 Medical Student Research Committee, Ahvaz Jundishapur University of Medical Sciences, Ahvaz, Iran

**Keywords:** occipital nerve block, migraine, lidocaine, triamcinolone

## Abstract

**Background:**

Despite a favorable clinical experience, little evidence exists for the efficacy of greater occipital nerve block (GONB) in migraine treatment. Considering such a premise, we wished to evaluate the therapeutic efficacy of GONB in patients affected by migraine headaches.

**Methods:**

A randomized double-blinded controlled trial was conducted on 48 patients suffering from migraine headaches. A syringe containing 1.0 mL of lidocaine 2%, 0.5 mL of either saline (control group, N = 24) or triamcinolone 0.5 mL (intervention group, N = 24) was prepared for each patient. Patients were assessed prior to the injection, and also 2 weeks, 1 month, and 2 months thereafter for severity and frequency of pain, times to use analgesics and any appeared side effects.

**Results:**

No significant differences were revealed in pain severity, pain frequency, and analgesics use between the two groups at the four study time points including at baseline, and 2, 4, and 8 weeks after the intervention. However, in both groups, the indices of pain severity, pain frequency, and analgesics use were significantly reduced at the three time points after the intervention compared with before the intervention.

**Conclusion:**

GONB with triamcinolone in combination with lidocaine or normal saline with lidocaine results in reducing pain severity and frequency as well as use of analgesics up to two months after the intervention, however any difference attributed to the drug regimens by assessing of the trend of pain characteristics changes.

## 1. Introduction

Migraine is now identified as one of the most frequent disabling medical conditions experienced worldwide that more than 90% of the patients report an impaired function during migraine attacks, and half of them report severe disability requiring bed rest (Goldstein et al., 1999; Kalra & Elliott, 2007; Estemalik & Tepper, 2013). The associated disabilities of migraine headaches result in spending indirect high costs due to decreased productivity and lost working days each year (Loder, Weizenbaum, Frishberg, & Silberstein, 2013). Various pharmacologic agents used as the treatment of migraine can be classified to abortive (for alleviating the acute phase) and prophylactic (Anonymous, 2004; Stephen & Silberstein, 2009; Silberstein et al., 2012; Koreshkina, 2014). Acute treatment including selective serotonin receptor agonists, ergot alkaloids, analgesics, non-steroidal anti-inflammatory drugs, alone or in combination with an anti-emetic are used to reverse, or at least stop progression of headache that is most effective when given within 15 minutes of pain onset. Preventive/prophylactic medications are administrated including antiepileptic drugs, beta blockers, tricyclic antidepressants, calcium channel blockers, selective serotonin reuptake inhibitors, serotonin antagonists, and even botulinum toxin. However, a subset of patients who neither achieve adequate pain relief nor can tolerate the side effects of typical migraine treatments emphasize requiring alternative medications (Cutrer & Charles, 2008; Kelley & Tepper, 2012). Peripheral nerve blocks have long been used in headache treatment. The most widely used procedure for this purpose has been greater occipital nerve (GON) block (Bovim et al., 1992). The rationale for using GONB in headache treatment comes from evidence of convergence of sensory input to trigeminal nucleus caudalis neurons from both cervical and trigeminal fibers (Sjaastad & Bakketeig, 2008). The GON is composed of sensory fibers that originate predominantly at the C2 level support the concept that the GON can be the irritable structure that generates occipital and fronto-orbital pain; pain which could be alleviated by anesthetic blocks and by neurolysis (Young, 2010).

Although there is no standardized procedure for GONB, the nerve is usually infiltrated by a local anesthetic or corticosteroid. Several studies suggested the efficacy of GON block in the treatment of migraine, cluster headache, and chronic daily headache. But, few of them were controlled and blinded. Despite a favorable clinical experience, little evidence exists for the efficacy of GON block in migraine treatment. Considering such a premise, we wished to evaluate the therapeutic efficacy of GON block in patients affected by migraine headaches.

## 2. Methods

### 2.1 Study Population

A randomized double-blinded controlled trial was conducted on 48 patients who suffering from migraine headaches and referred to neurology clinic of Golestan hospital in Ahvaz in 2013. These patients were considered to be included into the study by: age of 18 to 75 years, migraine history (with or without tenderness), at least one attack a week or MIDAS score greater than 11. The exclusion criteria were pregnancy or breast feeding, continuous headaches, opioid medications use, administration of prophylactic treatments for migraine within last two months, hypersensitivity to the drugs used in the study, local infection, cranial bone defect, chronic cluster headaches, chronic tension headaches, medication overuse headache, and fear from injection. According to inclusion criteria 52 patients entered into the study and 4 of them excluded because of not following the study protocol correctly or aggrevating headache without responding to GONB. This study was approved at 12/22/2012 by the Institutional Review Board of Studies in Human Subjects of ethics committee of Ahvaz JundiShapur University (Reference number: ETH-680). All patients provided a written informed consent prior to the enrollment.

### 2.2 Study Intervention

A syringe containing 1.0 mL of lidocaine 2%, 0.5 mL of either saline (control group, N = 24) or triamcinolone (intervention group, N = 24) was prepared for each patient. GONB were performed bilaterally. Using a 25 gauge needle, 1.5 mL was injected to each GON at the medial third of the distance between the occipital protuberance and the mastoid process. Patients were randomly assigned to either intervention or control groups and were blinded to the type of treatment they received. All patients were injected by a single physician who was not blinded to the type of treatment given. Demographic data were collected. Both groups received 20 mg tablets of Propranolol two times daily too. Patients were assessed prior to the injection, 2 weeks, 1 month, and 2 months after the injection by variables such as severity and frequency of pain, times to use analgesics and any appeared side effects. Headache severity was assessed on a 11 point scale. Analgesic use was measured as number of doses per week. Changes in symptom severity and other measured variables were compared between the two groups.

### 2.3 Statistical Analysis

Results were presented as mean ± standard deviation (SD) for quantitative variables and were summarized by frequency (percentage) for categorical variables. Continuous variables were compared using t test or Non-parametric Mann-Whitney U test whenever the data did not appear to have normal distribution or when the assumption of equal variances was violated across the two study groups. Categorical variables were, on the other hand, compared using chi-square test or Fisher’s exact test when more than 20% of cells with expected count of less than 5 were observed. The trend of the changes in study variables within study period was assessed using the Repeated Measure ANOVA test. For the statistical analysis, the statistical software SPSS version 21.0 for windows (SPSS Inc., Chicago, IL) was used. P values of 0.05 or less were considered statistically significant.

## 3. Results

The two intervention and control groups were matched in terms of mean age (37.00 ± 4.41 years versus 37.04 ± 9.93 years, p = 0.987), and female gender distribution (87.5% versus 91.7%, p = 0.999). The overall prevalence of occipital tenderness was 25.0% in intervention group and 20.8% in control group. As presented in Table 1, no significant differences were revealed in pain severity, pain frequency, and analgesics use at the four study time points including at baseline, and 2, 4, and 8 weeks after the intervention between the two groups. However, in both groups, the indices of pain severity, pain frequency, and analgesics use were significantly reduced two weeks after the intervention compared with before the intervention. Comparing these variables between the fourth week after the intervention and previous time points showed that all indices were significantly higher in the fourth week than the second week after the intervention, but the values of these three variables were significantly lower in the fourth week compared to baseline. Similar findings observed in the eighth week of the study, i.e. comparing these variables showed that although the variables were higher in the eighth week than the second and fourth week of intervention, but the values of these three variables were significantly lower in the eighth week compared to baseline. However, the trends of the changes in these variables were similar in the two groups within eight weeks of the study.

**Table 1:** Trend of the changes in pain severity, pain frequency, and time for analgesic use in intervention and control groups

Item	Baseline	Week 2	Week 4	Week 8	P (2,0)	P (4,0)	P (8.0)
Pain severity							
Intervention	7.46 ± 1.14	3.50 ± 0.44	4.29 ± 1.43	5.46 ± 1.24	< 0.001	< 0.001	< 0.001
Control	7.29 ± 1.04	3.50 ± 1.35	4.71 ± 1.27	5.29 ± 1.14	< 0.001	< 0.001	< 0.001
p-value	0.600	0.999	0.291	0.600	< 0.001	< 0.001	< 0.001
Pain frequency					< 0.001	< 0.001	< 0.001
Intervention	11.71 ± 1.03	5.50 ± 2.27	6.71 ± 2.22	8.38 ± 3.46	< 0.001	< 0.001	< 0.001
Control	11.36 ± 3.07	6.50 ± 1.82	7.58 ± 2.08	9.42 ± 3.83	< 0.001	< 0.001	< 0.001
p-value	0.734	0.099	0.165	0.328	< 0.001	< 0.001	< 0.001
Drug use					< 0.001	< 0.001	< 0.001
Intervention	11.17 ± 3.28	6.13 ± 1.65	7.29 ± 2.53	9.17 ± 3.28	< 0.001	< 0.001	< 0.001
Control	12.71 ± 2.97	6.25 ± 1.82	7.79 ± 2.23	10.71 ± 2.97	< 0.001	< 0.001	< 0.001
p-value	0.095	0.804	0.471	0.095	< 0.001	< 0.001	< 0.001

In addition to assessing changes in pain severity, pain frequency, and times to use analgesics in total subjects, the trends of the changes in these variables were also assessed in the patients with or without occipital tenderness in both intervention and control groups. As shown in Table 2 (for the intervention group) and in Table 3 (for the control group), no differences were found in the three study variables between those with or without tenderness at different time points of the study. However, the mean of these variables were significantly lower at three time points of 2, 4, and 8 weeks after the intervention compared with before it. Within the study period, no serious side effect was observed in both groups of the study.

**Table 2:** Trend of the changes in pain severity, pain frequency, and time for analgesic use in control group in patients with (n = 5) and without (n = 19) tenderness

Item	Baseline	Week 2	Week 4	Week 8	P (2,0)	P (4,0)	P (8.0)
Pain severity							
With tenderness	7.60 ± 1.14	3.60 ± 1.14	5.00 ± 0.71	5.60 ± 1.14	< 0.001	< 0.001	< 0.001
Without tenderness	7.21 ± 1.03	3.47 ± 1.43	4.63 ± 1.38	5.21 ± 1.03	< 0.001	< 0.001	< 0.001
p-value	0.515	0.840	0.425	0.515	< 0.001	< 0.001	< 0.001
Pain frequency					< 0.001	< 0.001	< 0.001
With tenderness	14.40 ± 1.34	7.40 ± 2.41	9.00 ± 2.12	1.00 ± 1.58	< 0.001	< 0.001	< 0.001
Without tenderness	11.58 ± 3.73	6.26 ± 1.63	7.21 ± 1.96	9.47 ± 3.70	< 0.001	< 0.001	< 0.001
p-value	0.067	0.364	0.140	0.242	< 0.001	< 0.001	< 0.001
Drug use					< 0.001	< 0.001	< 0.001
With tenderness	14.60 ± 1.34	7.00 ± 1.22	9.00 ± 1.22	12.60 ± 1.34	< 0.001	< 0.001	< 0.001
Without tenderness	12.21 ± 3.10	6.05 ± 1.93	7.47 ± 2.34	10.21 ± 3.10	< 0.001	< 0.001	< 0.001
p-value	0.111	0.208	0.069	0.111	< 0.001	< 0.001	< 0.001

**Table 3:** Trend of the changes in pain severity, pain frequency, and time for analgesic use in intervention group in patients with (n = 6) and without (n = 18) tenderness

Item	Baseline	Week 2	Week 4	Week 8	P (2,0)	P (4,0)	P (8.0)
Pain severity							
With tenderness	7.67 ± 0.52	3.50 ± 1.38	4.67 ± 1.23	6.00 ± 0.63	< 0.001	< 0.001	< 0.001
Without tenderness	7.39 ± 1.29	3.50 ± 1.50	4.17 ± 1.50	5.39 ± 1.29	< 0.001	< 0.001	< 0.001
p-value	0.461	0.999	0.429	0.281	< 0.001	< 0.001	< 0.001
Pain frequency					< 0.001	< 0.001	< 0.001
With tenderness	12.17 ± 2.32	5.83 ± 1.47	6.83 ± 1.83	8.67 ± 0.82	< 0.001	< 0.001	< 0.001
Without tenderness	11.56 ± 3.26	5.39 ± 2.50	7.67 ± 2.38	8.28 ± 4.00	< 0.001	< 0.001	< 0.001
p-value	0.625	0.605	0.862	0.701	< 0.001	< 0.001	< 0.001
Drug use					< 0.001	< 0.001	< 0.001
With tenderness	12.33 ± 3.72	6.17 ± 1.17	8.00 ± 3.22	10.33 ± 3.72	< 0.001	< 0.001	< 0.001
Without tenderness	10.78 ± 3.14	6.11 ± 1.81	7.06 ± 2.31	8.78 ± 3.14	< 0.001	< 0.001	< 0.001
p-value	0.325	0.932	0.529	0.386	< 0.001	< 0.001	< 0.001

## 4. Discussion

In the present study, the effect of GON blocking on migraine headaches were assessed by evaluating pain severity, pain frequency, and times to use analgesics. In this context, the changes in study variables were compared between the two groups including the intervention group which received triamcinolone and lidocaine and the control group received normal saline with lidocaine within eight weeks of study. Our study showed in both groups, a considerable decrease in three variables within two weeks from the injection time point in comparison to before the injection; however the trend of the changes in these variables were similar in both groups. On the other hand, our study showed that both drug regimens were similarly effective on reducing pain severity and frequency and on decrease of times to use an analgesic. Furthermore, the effects of these regimens persisted for 2 months after blocking of GON. Our findings were consistent with the previous studies. A research (Saracco et al., 2010) showed that adding triamcinolone to local anesthetic when performing GON blocks was not associated with improving outcome in the sample of patients with migraine, however in both groups, the procedure resulted in significant and rapid relief of headaches. Similar results were found in another study by Ashkenazi and colleagues (Ashkenazi et al., 2008). Saracco et al. (2010) showed that the anesthetic blockage with bupivacaine on the GON does not change the number of crises and their duration, but it provokes an intensity reduction after 60 days from the infiltration. Overally, it seems that the use of triamcinolone with lidocaine has no superiority on the use of lidocaine with normal saline for GON blocking, thus because of its probable side effects, administration of latter regimen is more recommended especially in repeated injections.

In other assessment aspect of the study, interactive effects of occipital tenderness on the efficacy of GON blocking in reducing severity and frequency of pain, was shown that the trend of improving of these variables were similar in those with or without occipital tenderness and thus the presence of tenderness could not interact with the therapeutic effects of study interventions. In other words, the presence of occipital tenderness has no significant role in the outcome of GON blocking in migraine headaches.

## 5. Conclusion

In conclusion, GON blocking with the two regimens including triamcinolone in combination with lidocaine or normal saline with lidocaine results in considerable reducing pain severity and frequency as well as use of analgesics up to two months after the intervention, although no significant differences attributed to the trend of changes of pain characteristics by one of the drug regimens. The presence of occipital tenderness has no effect on the changes of the trend of pain variables. According to our final findings, effects of the therapeutic interventions have been shown, but the superiority between one of the groups has not been indicated. Thus, in migraine status with MIDAS > 11 with no response or tolerance of common migraine treatments, GON blocking with lidocaine can be considered as a safe treatment approach.
